# Application of patient-reported outcome instruments in nonsurgical management of pelvic organ prolapse: a scoping review

**DOI:** 10.1590/1980-220X-REEUSP-2025-0341en

**Published:** 2026-07-31

**Authors:** Miao Li, Huan Wang, Yuanli Jia, Hangcheng Liu, Xixi Li

**Affiliations:** 1 University of Electronic Science and Technology of China, School of Medicine, Department of Nursing, Mianyang Central Hospital, Mianyang, Sichuan Province, China.; 2 North Sichuan Medical College, School of Nursing, Nanchong, Sichuan Province, China.

**Keywords:** Pelvic Organ Prolapse, Conservative Treatment, Patient Reported Outcome Measures, Prolapso de Órgãos Pélvicos, Tratamento Conservador, Medidas de Resultados Relatados pelo Paciente

## Abstract

**Objective:**

To review the content, characteristics, and current application status of patient-reported outcomes (PROs) for non-surgical treatment of pelvic organ prolapse (POP), providing information for future research.

**Methods:**

In accordance with the Preferred Reporting Items for Systematic Reviews and Meta-Analyses extension for scoping reviews (PRISMA-ScR) guidelines, a systematic search of PubMed, Web of Science, Embase, CINAHL, Cochrane Library, China National Knowledge Infrastructure (CNKI), Wanfang Database, VIP Database, and Chinese Biomedicine Literature Database (CBM-SinoMed) was conducted. The search period covered the inception of each database through January 16, 2025.

**Result:**

A total of 1548 studies were identified, of which 19 were included in the review. In the context of non-surgical POP, PROs were primarily used to evaluate clinical effectiveness, quality of life, and symptoms. Across the included literature, 29 PRO instruments were described, covering aspects such as patients’ physiological and psychological status, treatment satisfaction, and treatment goals.

**Conclusion:**

PROs application in non-surgical POP management remains limited. Future efforts should expand its scope, implement dynamic assessment across treatment stages, and utilize digital technology to improve the convenience and efficiency of assessment.

## RESUMO

**Objetivo:**

Revisar o conteúdo, as características e o status atual da aplicação dos resultados relatados pelo paciente (PROs) para o tratamento não cirúrgico do prolapso de órgãos pélvicos (POP), fornecendo informações para pesquisas futuras.

**Métodos:**

Em conformidade com a extensão das Diretrizes Preferred Reporting Items for Systematic Reviews and Meta-Analyses para revisões de escopo (PRISMA-ScR), uma busca sistemática no PubMed, Web of Science, Embase, CINAHL, Cochrane Library, China National Knowledge Infrastructure (CNKI), Wanfang Database, VIP Database e Chinese Biomedicine Literature Database (CBM-SinoMed) foi realizada. O período de pesquisa abrangeu o início de cada banco de dados até 16 de janeiro de 2025.

**Resultados:**

Um total de 1548 estudos foram identificados, dos quais 19 foram incluídos na revisão. No contexto do POP não cirúrgico, os PROs foram usados principalmente para avaliar a eficácia clínica, a qualidade de vida e os sintomas. Em toda a literatura incluída, 29 instrumentos de PRO foram descritos, abrangendo aspectos como o estado fisiológico e psicológico dos pacientes, a satisfação com o tratamento e os objetivos do tratamento.

**Conclusão:**

A aplicação de PROs no manejo não cirúrgico do POP permanece limitada. Esforços futuros devem expandir seu escopo, implementar a avaliação dinâmica em todas as fases do tratamento e utilizar a tecnologia digital para melhorar a conveniência e a eficiência da avaliação.

## INTRODUCTION

Pelvic organ prolapse (POP) is a condition characterized by the descent of one or more pelvic organs resulting from abnormalities in the pelvic floor muscles and fascia, leading to anatomical malposition and functional impairment. Its primary manifestation is a bulge or protrusion from the vagina, often accompanied by urinary, bowel, and sexual dysfunction^(1)^. The incidence rate of POP is as high as 30% globally, with a trend of increase year by year^(2)^. 50% of postpartum women will experience POP^(3)^. POP and its complications seriously affect the physical and mental health, social activities and quality of life of female patients, causing a huge economic burden to patients and their families, and increasing the difficulty of medical services and social pension^(4)^. With the deepening of the aging of the population, the disease has become a health problem of increasing concern for women around the world^(5)^. Treatment options for POP include surgical and non-surgical treatments. Compared with surgical intervention, non-surgical management offers the advantages of being non-invasive or minimally invasive and avoids surgery-associated risks, positioning it as the first-line treatment for POP^(6)^. Currently, the evaluation of the effectiveness of traditional non-surgical POP treatment is based on objective examination criteria, including the POP-Q examination or imaging, which ignores the patient’s own feelings and fails to comprehensively measure the impact of the disease and treatment on the patient’s health-related quality of life^(7)^. The International Consultation on Incontinence states^(8)^ that the most effective way to assess the perception and severity of pelvic floor prolapse symptoms and their impact on patients is to use Patient-Reported Outcomes (PROs). PROs refer to evaluations of one’s own health status and treatment outcomes that come directly from the patient, without interpretation by healthcare providers or others^(9)^. Patient reports are the key to patient-centered care, and the evaluation of their outcomes enables medical professionals to better understand the impact of disease and treatment on patients’ lives, contributing to improved quality of care and treatment satisfaction^(10)^. While prior systematic reviews have examined PRO instruments for POP^(11,12)^, they lack comprehensive analysis of clinical application trends and a standardized classification of tools for non-surgical management, hindering their effective selection and implementation in this population. Therefore, this study aims to review the content, characteristics, and current application status of PROs for non-surgical treatment of pelvic organ prolapse, aiming to establish a foundation for subsequent clinical research. The purpose of this scope review is to answer the following research questions: 1. What are the PRO instruments and contents used in non-surgical POP patients? 2. What are the characteristics of PRO instruments used in POP non-surgical therapy, including the form, frequency, timing, and field of application of the tools? 3. What are the problems and implications of using PROs for POP non-surgical patients?

## METHOD

### Study Design

The scoping review was based on the guidelines for scoping reviews as outlined in the Joanna Briggs Institute (JBI) manual for Evidence Synthesis^(13)^ and the Preferred Reporting Items for Systematic Reviews and Meta?Analyses (PRISMA) extension for scoping reviews (PRISMA-ScR)^(14)^. Based on the JBI guidelines the objectives, inclusion and exclusion criteria, search strategy, data extraction plan, and data analysis strategy were developed. To identify the main concept for the research questions and to provide a guide for the inclusion criteria, the population, concept, and context (PCC) framework was used^(13)^. We used the Preferred Reporting Items for Systematic Reviews and Meta?Analyses extension for Scoping Reviews (PRISMA? ScR) guidelines for the search strategy, data collection, and data reporting process.

### Inclusion Criteria

All articles in this scoping review met the following inclusion criteria: (a) women diagnosed with POP who are being or have been treated non-surgically^(15)^; (b) studies involving quantitative PROs outcomes in non-surgical treatment of POP; (c) patients receiving non-surgical treatment in the outpatient or at home. The following exclusion criteria were used: (a) reviews, guidelines, protocols, conference abstracts and papers, editorials, etc; (b) non-Chinese and English literature; (c) repeated publications, retracted literature; (d) Full text literature not available; (e) women who had surgery or planned to have surgery.

### Search Procedure

A systematic search was conducted in nine electronic databases, including PubMed, Web of Science, Embase, CINAHL, Cochrane Library, China National Knowledge Infrastructure (CNKI), Wanfang Database, VIP Database, and the Chinese Biomedicine Literature Database (CBM-SinoMed). The search covered the period from database inception to January 16, 2025. The search terms included “Pelvic Organ Prolapse/Urogenital Prolapse/Vaginal Vault Prolapse/Rectal Prolapse/Uterine Prolapse/Cystocele/Visceral Prolapse” “Patient Reported Outcome Measures/Patient reported outcome/patient health questionnaire/self-report”. The detailed search strategy for PubMed is presented in Table 1.

**Table 1:** Search strategies of PubMed databases – Mianyang, Sichuan, China, 2025.

PubMed	#1:”Pelvic Organ Prolapse”(MeSH Terms) OR “Pelvic Organ Prolapse”(Title/Abstract) OR “urogenital prolapse”(Title/Abstract) OR “vaginal vault prolapse”(Title/Abstract) OR “rectal prolapse”(Title/Abstract) OR “uterine prolapse”(Title/Abstract) OR “Cystocele”(Title/Abstract) OR “visceral prolapse”(Title/Abstract) #2:”Patient Reported Outcome Measures”(MeSH Terms) OR “Patient Reported Outcome Measures”(Title/Abstract) OR “patient reported outcome”(Title/Abstract) OR “patient health questionnaire”(Title/Abstract) OR “self-report”(Title/Abstract) #3: #1 ADN #2

### Literature Screening and Extraction

All retrieved articles were managed using EndNote 20. After removing duplicates, two researchers independently screened the titles and abstracts based on predefined eligibility criteria, followed by a full-text review. Any disagreements during the screening process were resolved through discussion with a third researcher. A standardized data extraction form was used to independently collect information, including author, publication year, country, sample size, study design, study population, PRO instrument, instrument content, application fields, assessment form, evaluation frequency, and assessment setting. The extracted data were then summarized and analyzed.

## RESULTS

### Literature Search

**Figure 1:**
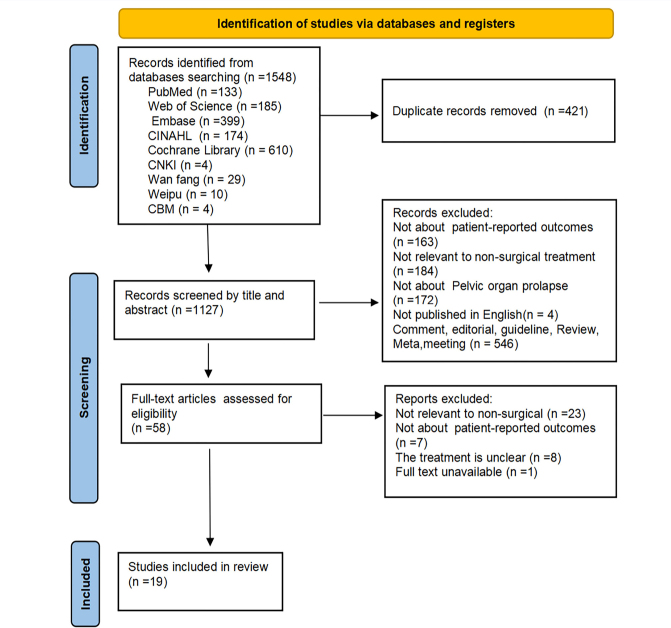
PRISMA flowchart of search strategy results – Mianyang, Sichuan, China, 2025.

The initial database search yielded 1548 articles, all of which were imported into EndNote 20. After duplicate removal, 1127 articles were retained. These articles were then screened by title and abstract, leading to the exclusion of 1069 articles. The remaining 78 articles underwent full-text screening, after which 39 were excluded. Ultimately, 19 studies met the inclusion criteria and were included in this review, as shown in Figure 1.

### Research Characteristics

The publication dates of the included literature range from 2014 to 2024, consisting of 18 English articles and 1 Chinese article. Currently, research in this field is geographically dispersed, primarily focused on developed countries, with a total of 11 articles published. Developing countries have published a total of 8 articles. There is variety in research design, mainly including 10 randomized controlled trials, 6 prospective studies, 2 quasi-experimental studies, and 1 retrospective case-control study. The basic characteristics of the included literature are shown in Table 2.

**Table 2:** Description of selected articles, according to author, year, country, study design, study population, sample form, size, PROs content, PRO instruments, application fields, assessment form ,evaluation frequency, assessment setting - Mianyang, Sichuan, China, 2025.

Author	Year	Country	Study design	Study population	Sample size	PROs content	PRO instruments	Application fields	Assessment form	Evaluation frequency	Assessment setting
Hagen et al.^(16)^	2016	UK, New Zealand	Multicenter randomized controlled trials	Women of any age with POP stage 1-3	412 (control group = 207/intervention group = 207)	Prolapse, sexual function, bladder and bowel symptoms, and quality of life	POP-SS PISQ4R SF-12 ICIQ-UI-SF ICIQ-B	Clinical effectiveness evaluation	Mailed paper questionnaires	Baseline, 1 and 2 years after randomization grouping	Gynecology clinics in participating centers
Hagen et al.^(17)^	2014	UK, New Zealand, Australia	Multicenter randomized controlled trials	Female with stage 1-3 prolapse	447 (control group = 22/intervention group = 225)	Prolapse, sexual function, bladder and bowel symptoms, and quality of life	POP-SS PISQ-IR SF-12 ICIQ-UI-SF ICIQ-B	Clinical effectiveness evaluation	Mail paper or phone	Baseline, 6 and 12 months after enrollment	Gynecology clinics in participating centers
Strohbehn et al.^(18)^	2024	United States	Prospective studies	Patients with POP-Q stage ≥2 prolapse	78	Prolapse, sexual function, bladder, bowel symptoms, pain, satisfaction	PFDI-20 PFIQ-7 PISQ-IR Global satisfaction VAS	Clinical effectiveness evaluation	Face-to-face paper or telephone	1 month at baseline and 3 months in the intervention period (visits 0, 1 and 3)	Urogynecology clinical trial site
Mao et al.^(l9)^	2018	China	Prospective studies	Patient successfully wearing a supported ring uterine tray	142	Prolapse, bladder, bowel symptoms, body imagery	PFDI-20 PFIQ-7 PGI-C	Symptom assessment/Quality of life	Face-to-face paper or telephone	Median follow-up of 17 months (range 13-24 months), follow-up time points (months 3, 6, 12, and every 6 months thereafter)	Hospital outpatient clinics for obstetrics and gynecology
Bugge et al.^(20)^	2024	Britain	Randomized controlled trial	Patients using uterine supports for at least two weeks	340 (control 169/intervention group 171)	Pelvic floor symptoms (prolapse, bladder, bowel), sexual function, quality of life, self-efficacy	PFIQ-7 PFDI-20 EQ-5D-5L GSES PISQ-IR PGI-I	Clinical effectiveness evaluation/Quality of life	Mailed paper, telephone, face-to-face, electronic links	Total 18 months, baseline, follow-up 6 months, 12 months, 18 months	Hospital maternity or clinic outpatient
Meriwether et al.^(21)^	2015	United States	Prospective studies	Patients with uterine supports in place	127	Sexual Function, Body Imagery	PISQ-IR mBIS	Quality of life	Face-to-face paper	Baseline, three months	Uterine tray management clinic
Ai et al.^(22)^	2018	China	Prospective studies	Patients using uterine supports	102	Prolapse, Bowel, Bladder, Quality of Life, Depression	PFDI-20 PFIQ-7 PHQ-9	Symptom assessment/Quality of life	Face-to-face paper	Baseline, 3 months	Pelvic Floor Center Clinic
Ouch et al.^(23)^	2018	Japan	Quasi-experi mental studies	Patients with stage 2 or 3 prolapse	27	Quality of life	P-QOL POP-specific QOL	Quality of life/Symptom assessment	Face-to-face paper or telephone	Baseline (pre-PFMT), at the end of 4-month PFMT, 2-year follow-up	Urological Surgery Department to or from home
Gorji et al.^(24)^	2019	Iran	Randomized Controlled Trial	Women with POP-Q stage 2 or 3	40 (20 in the intervention group/20 in the control group)	Lower urinary tract symptoms, quality of life	ICIQ-FLUTS P-QOL	Clinical effectiveness evaluation	Unreported	Baseline, 4 weeks postintervention	Urology Clinic
Pizarro-Berdichevsky et al.^(25)^	2016	Chile	Prospective studies	Patients with stage 2 and above	91	Quality of life, depression	PFDI-20 P-QOL PISQ-12 GHQ-12	Symptom assessment/Quality of life	Unreported	Baseline, after 12 months of follow-up	Urogynecology Clinic
Limbutara et al.^(26)^	2023	Thailand	Randomized Controlled Trial	Women with POP-Q stage 2 or 3	40	Quality of life, sexual function, treatment goals	GAS VAS for Treatment Goal Achievement PISQ-IR P-QOL	Clinical effectiveness evaluation	Face-to-face paper	Baseline, 2 weeks, 6 weeks posttreatment	Urogynecology Clinic
Crowle and Harley(^27)^	2020	Britain	Retrospective casecontrol study	Conservatively treated patients	23	Prolapse, bladder, bowel symptoms	PFDI-20	Clinical effectiveness evaluation	Face-to-face paper	Baseline, after last treatment (median treatment duration 3 months)	Private hospitals or physiotherapy clinics
Sweta et al.^(28)^	2018	India	Randomized Controlled Trial	Patients with mild POP	50 (intervention group 25/control group 25)	Prolapse, bladder, bowel symptoms	PFDI-20 PFIQ-7	Clinical effectiveness evaluation	Face-to-face paper	Baseline, postintervention weeks 4, 8, and end of 12	unreported
Shobeiri and Santiago(^29)^	2014	United States	Randomized Controlled Trial	Patients with stages 1 to 3 prolapse	447 (intervention group 225/control group 222)	Prolapse Symptoms	POP-SS	Clinical effectiveness evaluation	Unreported	At baseline, 6 and 12 months	unreported
Wiegersma et al.^(30)^	2014	Netherlands	Randomized Controlled Trial	Stage 1 and 2 elderly patients	287 (intervention group 145/control group 142)	Perception of prolapse, bladder, bowel, sexual function, quality of life, symptomatic changes	PFDI-20 PFIQ-7 PISQ-12 SF-12 VAS for Treatment Effect Evaluation	Symptom assessment	Face-to-face paper/mail questionnaire	Baseline, 3 months postintervention	Unreported
Wiegersma et al.^(31)^	2014	Netherlands	Randomized Controlled Trial	Stage 2 and 3 elderly patients	447 (intervention group 226/control group 221)	Prolapse, bladder, bowel, sexual dysfunction, quality of life, symptom burden	PFDI-20 PFIQ-7 PISQ-12 SF-12 EQ-5D	Clinical effectiveness evaluation	Face-to-face paper/mail questionnaire	Baseline, 3 months, 12 months, 24 months after treatment	Unreported
Min et al.^(32)^	2023	China	Randomized Controlled Trial	Patients with stages 1-3 prolapse	117 (control group 58/intervention group 59)	Prolapse, bladder, bowel, sexual function, quality of life, anxiety, depression, self-efficacy	PFDI-20 PISQ-12 SAS SDS QLQ-52 GSES	Clinical effectiveness evaluation	Face-to-face paper	Baseline, 3 months postintervention	Hospital outpatient clinics
Ziv and Erlich(^33)^	2022	Israel	Quasi-experi mental study	Women with POP-Q stage 2-4 prolapse	52	Prolapse, bladder, bowel, quality of life	PFDI-20 PFIQ-7 POP symptoms alleviation score	Quality of life/Clinical effectiveness evaluation	Face-to-face paper or telephone	Baseline, 2 years after use	Clinic visits
Coelho et al.^(34)^	2018	Brazil	Prospective studies	Patients with stage 3-4 prolapse	19	Vaginal symptoms, sexuality, quality of life	SF-36 ICIQ-VS	Quality of life/Symptom assessment	Face-to-face paper	Baseline, 6 months postuse	Gynecology clinic

PFDI-20: Pelvic Floor Distress Inventory-20; PFIQ-7: Pelvic Floor Impact Questionnaire-7; PISQ-IR: Pelvic Organ Prolapse/Urinary Incontinence Sexual Questionnaire; ICIQ-UI-SF: ICIQ-Urinary Incontinence Short Form; ICIQ-FLUTS: International Consultation on Incontinence Questionnaire-Female Lower Urinary Tract Symptoms; ICIQ-B: International Consultation on Incontinence Questionnaires-Bladder; PISQ-12: Incontinence Sexual Function Questionnaire-12; ICIQ-VS: International Consultation on Incontinence Questionnaire-Vaginal Symptoms; POP-SS: Pelvic Organ Prolapse Symptom Score; POP symptoms alleviation score: the self-administered Pelvic Organ Prolapse Score; POPspecific QOL: self-administered Prolapse Symptom Report; P-QOL: Prolapse-quality of life questionnaire; SF-12: 12-ltem Short-Form Survey; QLQ-52: Quality of life-52; SF-36: MOS 36-ltem Short-Form Health Survey; EQ-5D-5L: EuroQol-5 Dimensions, five-level; EQ-5D: EuroQol-5D questionnaire; SAS: Self-Rating Anxiety Scale; SDS: Self-Rating Depression Scale; PHQ-9: Patient Health Questionnaire-9; GHQ-12: 12-item Goldberg Health Questionnaire; VAS: Visual Analogue Scale; mBIS: Modified female body image scale; CAS: Goal Attainment Scaling; PGI-I: Patient Global Impression of Improvement; PGI-C: Patient Global Impression of Change; GSES: General Self-efficacy Scale.

### PRO Instrument and Content

A total of 29 PRO instruments applied in the non-surgical management of POP were identified in the 19 included studies. The target population of these tools was predominantly patients with POP-Q stage I-III prolapse, and the domains assessed across all instruments included symptom assessment, psychological status, quality of life, and goal attainment. For symptom assessment, the instruments used included the Pelvic Floor Distress Inventory-20 (PFDI-20), Pelvic Floor Impact Questionnaire-7 (PFIQ-7), Pelvic Organ Prolapse/Urinary Incontinence Sexual Questionnaire (PISQ-IR), ICIQ-Urinary Incontinence Short Form (ICIQ-UI-SF), International Consultation on Incontinence Questionnaire-Female Lower Urinary Tract Symptoms (ICIQ-FLUTS), International Consultation on Incontinence Questionnaire-Bladder (ICIQ-B), Incontinence Sexual Function Questionnaire-12 (PISQ-12), Pelvic Organ Prolapse Symptom Score (POP-SS), a self-administered Pelvic Organ Prolapse Score (for symptom alleviation), a self-administered Prolapse Symptom Report, and the International Consultation on Incontinence Questionnaire-Vaginal Symptoms (ICIQ-VS). For the assessment of quality of life, the identified instruments were the Prolapse Quality of Life Questionnaire (P-QOL), 12-Item Short-Form Survey (SF-12), Quality of Life Questionnaire-52 (QLQ-52), 36-Item Short-Form Health Survey (SF-36), EuroQol-5 Dimensions-5 Level (EQ-5D-5L), and the EuroQol-5D questionnaire (EQ-5D). Regarding psychological status, the scales used included the Self-Rating Anxiety Scale (SAS), Self-Rating Depression Scale (SDS), Patient Health Questionnaire-9 (PHQ-9), 12-item Goldberg Health Questionnaire (GHQ-12), and the modified Female Body Image Scale (mBIS). Finally, tools related to goal attainment and treatment satisfaction comprised Goal Attainment Scaling (GAS), a Visual Analogue Scale for Treatment Goal Achievement, the Patient Global Impression of Improvement (PGI-I), Patient Global Impression of Change (PGI-C), the General Self-Efficacy Scale (GSES), and a Visual Analogue Scale for Global Satisfaction.

### Features of Instrument

Among the 29 PRO instruments included in this study, except for 2 PRO instruments that have no clear dimensional distinction, the other PRO instruments are composed of 1 to 9 dimensions. The number of items per tool varied widely, ranging from 1 to 52, and the primary scoring method was the Likert scale. Most of the included studies were follow-up investigations requiring patients to adhere to long-term treatment. Only a small subset of patients receiving self-management did not need to attend hospital for in-person assessments by medical staff during follow-up. Consequently, data collection primarily took place in hospital outpatient departments, physical therapy centers, and community settings. In the form of questionnaire collection, paper questionnaire collection is still the main form. Among the 19 studies, 3 studies^(24,25,29)^ failed to report in detail the form of PROs questionnaire collection, and 8 studies^(16,22,26,27,28,31,32,34)^ used a single paper questionnaire collection method. The remaining 8 studies employed a combination of methods, including telephone and mail questionnaires. Notably, only one study^(20)^ used electronic questionnaires. This study also mentioned providing contact information and multiple response formats to accommodate patients’ preferences, thereby offering a more flexible assessment approach. A total of 17 studies^(16,17,18,19,20,21,22,23,24,25,26,28,30,31,32,33,34)^ used multiple PRO instruments simultaneously to measure patients’ symptoms, quality of life, satisfaction, and psychological status. The other 2 studies^(27,29)^ with the theme of improving prolapse symptoms used a single PROs for singleattribute measurement of the severity of patients’ symptoms, but 1 of these studies had the tool itself as a multi-attribute measurement tool, which was able to reflect the severity of patients’ symptoms, quality of life, etc.

## APPLICATION FIELDS OF PRO INSTRUMENTS

### Clinical Effectiveness Evaluation

The International Advisory Committee on Urogynecology recommends^(35)^ that the validated PROs should be used as the gold standard in evaluating the symptoms of POP, together with quantitative clinical pelvic organ prolapse (POP-Q) examination. Therefore, the use of PROs as an evaluation indicator for the clinical treatment of patients is one of the most important application directions in the field of non-surgical treatment of POP. Of the 19 studies included, 12 involved the use of PROs as an outcome measure for the evaluation of treatment outcomes. These studies mostly focused on patients with POP-Q stage I-III prolapse and evaluated patient health management in home or outpatient settings, as well as the effects of various non-surgical interventions. Measures evaluated included personalized pelvic floor exercises, the use of novel pessaries, the implementation of nursing models based on self-efficacy theories, and physical exercise activities such as yoga and postures. The indicators evaluated mainly focused on POP related symptoms, including prolapse, abnormal urination, abnormal bowel movements, and sexual disturbance. In addition, other indicators were also involved in the study, such as patients’ satisfaction with the treatment effect, treatment confidence, completion of treatment goals, and compliance.

### Quality of Life

Although POP is a non-fatal condition, it has a high incidence rate, substantial treatment costs, and significantly affects the physical and mental health and quality of life of affected women^(36)^. Health-related quality of life, which refers to an individual’s overall health status and subjective well-being across physical, mental, and social domains, is a key construct assessed by PRO instruments in various healthcare-related contexts^(37)^. Therefore, using PRO tools to evaluate quality of life in patients undergoing non-surgical POP treatment represents another important research focus in this field. 8 of the 19 studies^(19,20,21,22,23,25,33,34)^ assessed patients’ quality of life. Most studies used generic quality of life instruments for pelvic floor disorders, while only five^(23,24,25,26,34)^ employed the P-QOL scale, a POP-specific questionnaire. In the remaining studies, although quality of life was not the primary focus, the PRO instruments used included domains related to this construct. This reflects a broader effort to comprehensively understand how non-surgical

treatments improve daily functioning and overall well-being in patients with POP.

### Symptom Assessment

The primary goal of POP treatment is to alleviate symptoms. Therefore, using PRO instruments to evaluate and manage physical and psychological symptoms or functioning during non-surgical treatment is a key area of research. Of the 19 included studies, 6^(14,22,23,25,30,34)^ assessed mental and physical functioning in patients undergoing non-surgical treatment.

These studies evaluated a wide range of symptoms across diverse domains. Three studies^(19,23,34)^ focused exclusively on physical symptoms, with bulging and pelvic pressure being the most commonly reported. Additionally, three studies^(22,25,30)^ assessed physical symptoms alongside other outcomes, rather than focusing on them as primary endpoints. The use of PRO instruments to assess psychological symptoms in POP patients was also a key research focus. Three studies^(18,22,25)^ assessed psychological symptoms, a relatively small number. However, all were large prospective studies, with two^(22,25)^ specifically examining the causal relationship between depressive symptoms and POP. Other psychological symptoms assessed included anxiety, distress, and related functional impairments.

### PRO Instruments Assessment Time

Each of the 19 articles included in this study reported the evaluation time points of PRO instruments in patients with POP receiving nonsurgical treatment. Although the time points for PROs data collection varied across studies, all included studies performed a baseline assessment before the intervention or treatment initiation. The number of questionnaire assessments per patient ranged mainly from 2 to 5 times. The majority of studies^(21,22,24,25,27,30,32,34)^ conducted two assessments, consisting of PROs questionnaire completion before and after nonsurgical intervention. In addition, 5 studies^(19,20,23,28,31)^ performed PROs assessments more than 3 times. All these were follow-up studies with a relatively long follow-up period, ranging from 2 weeks to 2 years, and the number of assessments increased with longer follow-up duration. Among them, PROs data collection time points were mainly concentrated at 3, 6, and 12 months, which are widely used to evaluate the medium- and long-term effects of interventions. Some studies were designed to monitor changes in treatment outcomes during home-based followup among patients receiving nonsurgical management. These studies had flexible designs and variable follow-up durations, which may include pre- and post-intervention assessments as well as periodic follow-up assessments.

## DISCUSSION

### Great Heterogeneity of PRO Instruments in the Field of Non-Surgical Treatment of POP

Currently, wide variations exist in the types of PROs, content, number of items, and assessment time points used in the field of non-surgical POP treatment. A wide variety of PROs are available for assessing symptoms in patients undergoing non-surgical POP treatment. Among those included in this review, the most frequently used were the PFDI-20,

PFIQ-7, and PISQ-IR. However, due to the limitations in language and cross-cultural adaptation, the validation and application rates of some scales remain low in different countries and regions^(11)^. This to some extent hinders the promotion of PRO tools in the international field of POP non-surgical treatment and the comparison of high-quality cross-national studies. In addition, 2 studies^(23,33)^ used self-designed symptom assessment questionnaires for non-surgical treatment of POP patients, but the procedures and steps of questionnaire preparation were not reported in the studies, and their reliability, validity and representativeness need to be further verified. Therefore, future efforts should emphasize the rigorous translation, cross-cultural adaptation, and validation of existing tools. Second, the 29 PROs included in this review varied considerably in their dimensional content, covering multiple domains. In terms of assessment priorities for POP patients, Sung et al.^(38)^ outlined key constructs, including (1) relief of vaginal bulge symptoms, (2) improvement in physical functioning, (3) improvement in sexual functioning, (4) improvement in body image perception, and (5) improvement in social functioning. However, none of the PROs included in this review covered all of these constructs, limiting the comprehensiveness of assessments in patients undergoing non-surgical POP treatment. In addition, the choice of PRO assessment time points varied across studies. 11 studies assessed patients at baseline, post-intervention, and follow-up, with intervals ranging from weeks to years. Four studies^(18,19,23,31)^ employed a stage-stratified approach to data collection. The heterogeneity in assessment time points may have contributed to variability in study findings. Previous research^(35)^ has noted a lack of longitudinal data on the progression, resolution, and treatment of POP. Consequently, the clinical importance of early intervention and the optimal timing of treatment remain unclear. The variability in PRO assessment timing may be one factor hindering a comprehensive understanding of symptom trajectories and treatment outcomes in non-surgical POP patients. Additionally, some studies administered multiple PROs concurrently, leading to an increased number of items or conceptual overlap, thereby increasing the burden on patients^(39)^. Therefore, future efforts should focus on developing comprehensive and concise PRO tools for non-surgical POP patients, to accurately capture patient characteristics and needs. At the same time, there is an urgent need to establish a standardized assessment timeline based on the characteristics of different PRO tools, thereby improving the PRO evaluation system for non-surgical POP patients.

### The PROs Digital Health Monitoring Model in the Field of Non-Surgical Treatment of POP Needs to be Improved

PRO instruments are typically completed by patients and can be administered through various formats, including paperbased questionnaires, telephone interviews, mailed questionnaires, and electronic surveys. Different PRO formats require separate validation^(40)^. The findings indicate that current PRO data collection in non-surgical POP treatment primarily relies on paper-based questionnaires alone or a combination of telephone and mail questionnaires. Previous studies^(41)^ have shown that traditional paper-based data collection methods are not only less efficient but also prone to issues such as incomplete data and information loss. In this review, only Bugge et al.^(20)^ used electronic questionnaires to collect PRO data. However, the study did not perform further psychometric validation of the electronic version, which may affect the reliability and validity of its findings. Gray et al.^(42)^ found that electronic PRO measures (ePROs) can reduce researcher burden in data collection and management, improve data processing efficiency and accuracy, and allow patients to complete assessments at home prior to their initial visit. This enables clinicians to access key patient information in advance, thereby optimizing the diagnostic and treatment process. In addition, ePAQ is cost-effective, enabling faster data collection and significantly reducing both management time and implementation costs^(43)^. Currently, the ePAQ-Pelvic Floor (ePAQ-PF) is the only well-validated POPspecific electronic questionnaire^(44)^. However, it has not been used in patients receiving exclusively non-surgical treatment for POP and was therefore not included in this review. Therefore, future research should focus on developing electronic PRO tools specifically for non-surgical POP patients, integrating various digital health technologies to enhance patient experience and address diverse research needs. In addition, when converting existing paper questionnaires to electronic formats, it is essential to verify their psychometric properties to ensure the validity and reliability of the new versions.

### The Timeliness of PROs to Evaluate the Dynamic Changes in Symptoms of POP Non-Surgical Patients Still Needs to be Strengthened

As healthcare shifts toward a holistic, patient-centered model, the use of PROs in patients undergoing non-surgical treatment for POP is becoming increasingly important^(45)^. Studies have demonstrated that PROs should be incorporated into daily clinical practice and research, as they facilitate communication between patients and clinicians and support the development of optimal treatment strategies^(46)^. This review found that PRO tools are primarily used in non-surgical POP patients to assess symptoms, quality of life, and the clinical effects of non-surgical interventions. Research on symptom assessment remains limited, focusing primarily on post-intervention symptom improvement or correlations between POP and other symptoms, with little attention to long-term tracking of dynamic symptom changes. However, studies have shown^(47)^ that symptoms in POP patients are not static but fluctuate over time. Some of the studies included in this review^(22,25,30,34)^ focused only on symptom changes at baseline and follow-up endpoints, overlooking dynamic symptom fluctuations. This may have led researchers to overestimate or underestimate intervention efficacy and hindered in-depth analysis of the mechanisms linking POP symptoms and related conditions. In addition, most patients prefer flexible assessment timing when using PRO tools, allowing them to provide timely feedback to healthcare providers and receive targeted interventions when unwell^(48)^. Existing studies have primarily focused on structured PRO reporting at fixed intervals during POP treatment and follow-up^(49)^. However, this approach may lead to under-reporting or delayed symptom reporting, thereby diminishing the value of PROs for dynamic monitoring. Therefore, future studies should pay greater attention to dynamic symptom changes in patients undergoing non-surgical POP treatment, in order to more fully capture patients’ realtime experiences and provide a more accurate basis for clinical decision-making. At the same time, optimizing the assessment process and enhancing real-time patient monitoring represent important directions for future research in this field.

### PROs Report Results Have Gradually Become an Important Outcome Indicator

The National Institute for Health and Clinical Excellence (NICE)^(50,51)^ in the UK recommends non-surgical treatment as the first-line option for all patients with POP. Therefore, measuring outcomes of non-surgical treatment is essential for subsequent patient management. Previous studies have primarily evaluated treatment outcomes in POP patients using clinical objective indicators. However, these indicators have limitations in assessing the effectiveness of treatment outcomes. On one hand, studies^(52,53)^ have shown that anatomical changes in POP do not always correlate with symptom severity. Consequently, while objective indicators such as organ position and symptomatic staging may improve after treatment, patients’ perceptions of symptoms, quality of life, or treatment satisfaction may not improve concurrently. On the other hand, factors such as patients’ daily experiences, psychological state, and social functioning can significantly influence their disease experience and coping ability. These dynamic, multi-dimensional subjective perceptions cannot be fully captured by objective methods such as pelvic examination or imaging^(38)^. Thus, PROs are crucial in evaluating non-surgical POP treatment and reflect a patientcentered approach to care. The studies included in this review used quantitative PRO tools to report outcomes in patients undergoing non-surgical POP treatment. Among the 12 studies that assessed clinical outcomes, eight used PRO results as their primary outcome measure^(16,17,18,20,24,26,29,31)^. Experts in the pelvic floor field and relevant committees^(54)^ advocate for including PRO results in POP treatment outcomes, a recommendation consistent with the findings of this review. Finally, it should be noted that patient-reported health outcome data from PROs are an important component of clinical outcomes. However, PRO results should be integrated with clinical laboratory indicators, clinician-reported outcome measures (ClinROMs), and other outcome data from multiple sources to comprehensively reflect patient health and evaluate the effects of clinical interventions^(55)^.

## CONCLUSION

PROs have been used in the non-surgical treatment of POP to evaluate clinical outcomes, quality of life, and symptom assessment, mainly to measure the degree of improvement in patients’ symptoms, including the evaluation of patients’ quality of life and psychological symptoms. However, some studies

have not tracked the dynamic changes in patients’ symptoms, resulting in a lack of individualized supervision and management of patients. In the future, uniform standards for assessment frequency and interval should be set, and a dynamic tracking mechanism should be established to better monitor patients’ symptomatic changes, while digital technology should be used to improve the convenience and efficiency of assessment. In addition, the application fields of PROs should be expanded and the comprehensive assessment of patients’ psychological status should be strengthened, and multidisciplinary cooperation and international collaboration should be vigorously enhanced to promote the development of individualized management mode based on PROs. However, a key limitation of this review is the exclusion of non-English and non-Chinese studies. This decision may have limited the generalizability of our findings and introduced a language bias. Future reviews should therefore consider including a broader range of languages to ensure a more comprehensive and culturally diverse understanding of PRO applications in POP treatment.

## Data Availability

The entire dataset supporting the results of this study was published in the article itself.
