# Supplementary material for: Application of patient-reported outcome instruments in nonsurgical management of pelvic organ prolapse: a scoping review
Source: Rev Esc Enferm USP. 2026 Jul 31;60:e20250341. doi: 10.1590/1980-220X-REEUSP-2025-0341en (PMC13427262; doi:10.1590/1980-220X-REEUSP-2025-0341en)
Supplement: Translation

# Translation: Aplicación de instrumentos de resultados informados por los pacientes en el tratamiento no quirúrgico del prolapso de órganos pélvicos: una revisión de alcance

**DOI:** 10.1590/1980-220X-REEUSP-2025-0341es

**Authors:** Miao Li, Huan Wang, Yuanli Jia, Hangcheng Liu, Xixi Li

**Affiliations:** 1 University of Electronic Science and Technology of China, School of Medicine, Department of Nursing, Mianyang Central Hospital, Mianyang, Sichuan Province, China.; 2 North Sichuan Medical College, School of Nursing, Nanchong, Sichuan Province, China.

**Keywords:** Prolapso de Órganos Pélvicos, Tratamiento Conservador, Medición de Resultados Informados por el Paciente

## Abstract

**Objetivo:**

Revisar el contenido, las características y el estado actual de la aplicación de los resultados reportados por los pacientes (PRO, por sus siglas en inglés) para el tratamiento no quirúrgico del prolapso de órganos pélvicos (POP), proporcionando información para futuras investigaciones.

**Método:**

De acuerdo con las directrices de la extensión de elementos de informe preferidos para revisiones sistemáticas y metaanálisis para revisiones de alcance (PRISMA-ScR), se realizó una búsqueda sistemática en PubMed, Web of Science, Embase, CINAHL, Cochrane Library, China National Knowledge Infrastructure (CNKI), Wanfang Database, VIP Database y Chinese Biomedicine Literature Database (CBM-SinoMed). El período de búsqueda abarcó desde el inicio de cada base de datos hasta el 16 de enero de 2025.

**Resultados:**

Se identificaron un total de 1548 estudios, de los cuales 19 se incluyeron en la revisión. En el contexto del POP no quirúrgico, los PRO se utilizaron principalmente para evaluar la eficacia clínica, la calidad de vida y los síntomas. En toda la literatura incluida, se describieron 29 instrumentos PRO, que cubrían aspectos como el estado fisiológico y psicológico de los pacientes, la satisfacción con el tratamiento y los objetivos del tratamiento.

**Conclusión:**

La aplicación de los PRO en el manejo no quirúrgico del POP sigue siendo limitada. Los esfuerzos futuros deben ampliar su alcance, implementar una evaluación dinámica en todas las etapas del tratamiento y utilizar la tecnología digital para mejorar la conveniencia y la eficiencia de la evaluación.

## INTRODUCCIÓN

El prolapso de órganos pélvicos (POP) es una afección caracterizada por el descenso de uno o más órganos pélvicos como resultado de anomalías en los músculos y la fascia del suelo pélvico, lo que conduce a una mala posición anatómica y a un deterioro funcional. Su manifestación principal es un abultamiento o protrusión desde la vagina, a menudo acompañado de disfunción urinaria, intestinal y sexual^(1)^. La tasa de incidencia del POP alcanza el 30% a nivel mundial, con una tendencia al alza año tras año^(2)^. El 50% de las mujeres posparto experimentarán POP^(3)^. El POP y sus complicaciones afectan gravemente la salud física y mental, las actividades sociales y la calidad de vida de las pacientes, lo que genera una enorme carga económica para ellas y sus familias, y aumenta la dificultad de los servicios médicos y las prestaciones sociales^(4)^. Con el avance del envejecimiento de la población, la enfermedad se ha convertido en un problema de salud que preocupa cada vez más a las mujeres de todo el mundo^(5)^. Las opciones de tratamiento para el POP incluyen tratamientos quirúrgicos y no quirúrgicos. En comparación con la intervención quirúrgica, el manejo no quirúrgico ofrece las ventajas de ser no invasivo o mínimamente invasivo y evita los riesgos asociados a la cirugía, lo que lo posiciona como el tratamiento de primera línea para el POP^(6)^. Actualmente, la evaluación de la eficacia del tratamiento tradicional no quirúrgico del POP se basa en criterios de examen objetivos, como el cuestionario POP-Q o las pruebas de imagen, lo que ignora los propios sentimientos de la paciente y no mide de manera integral el impacto de la enfermedad y el tratamiento en la calidad de vida relacionada con la salud de la paciente^(7)^. La Consulta Internacional sobre Incontinencia afirma^(8)^ que la forma más eficaz de evaluar la percepción y la gravedad de los síntomas del prolapso del suelo pélvico y su impacto en las pacientes es utilizar los resultados informados por la paciente (Patient-Reported Outcomes - PRO). Los PRO se refieren a las evaluaciones del propio estado de salud y de los resultados del tratamiento que provienen directamente de la paciente, sin interpretación por parte de los profesionales de la salud u otras personas^(9)^.

Los informes de los pacientes son la clave de la atención centrada en el paciente, y la evaluación de sus resultados permite a los profesionales médicos comprender mejor el impacto de la enfermedad y el tratamiento en la vida de los pacientes, lo que contribuye a mejorar la calidad de la atención y la satisfacción con el tratamiento^(10)^. Si bien en revisiones sistemáticas anteriores se han examinado instrumentos de PRO para el POP^(11,12)^, carecen de un análisis exhaustivo de las tendencias de aplicación clínica y de una clasificación estandarizada de las herramientas para el manejo no quirúrgico, lo que dificulta su selección e implementación efectivas en esta población. Por lo tanto, este estudio tiene como objetivo revisar el contenido, las características y el estado actual de la aplicación de los PRO para el tratamiento no quirúrgico del prolapso de órganos pélvicos, con el fin de establecer una base para la investigación clínica posterior. El propósito de esta revisión de alcance es responder a las siguientes preguntas de investigación: 1. ¿Cuáles son los instrumentos y contenidos de los PRO utilizados en pacientes con POP no quirúrgico? 2. ¿Cuáles son las características de los instrumentos de PRO utilizados en la terapia no quirúrgica del POP, incluyendo la forma, la frecuencia, el momento y el campo de aplicación de las herramientas? 3. ¿Cuáles son los problemas y las implicaciones del uso de los PRO para pacientes con POP no quirúrgico?

## MÉTODO

### Diseño del Estudio

La revisión de alcance se basó en las directrices para revisiones de alcance descritas en el manual del Instituto Joanna Briggs (JBI) para la síntesis de evidencia^(13)^ y en la extensión de los Elementos de informe preferidos para revisiones sistemáticas y metaanálisis (PRISMA) para revisiones de alcance (PRISMA-ScR)^(14)^. A partir de las directrices del JBI, se desarrollaron los objetivos, los criterios de inclusión y exclusión, la estrategia de búsqueda, el plan de extracción de datos y la estrategia de análisis de datos. Para identificar el concepto principal de las preguntas de investigación y proporcionar una guía para los criterios de inclusión, se utilizó el marco de población, concepto y contexto (PCC)^(13)^. Utilizamos las directrices de la extensión de los Elementos de informe preferidos para revisiones sistemáticas y metaanálisis para revisiones de alcance (PRISMA-ScR) para la estrategia de búsqueda, la recolección de datos y el proceso de presentación de datos.

### CRITERIOS DE INCLUSIÓN

Todos los artículos de esta revisión de alcance cumplieron los siguientes criterios de inclusión: (a) mujeres diagnosticadas con POP que están siendo o han sido tratadas de forma no quirúrgica^(15)^; (b) estudios que incluyan resultados cuantitativos de PRO en el tratamiento no quirúrgico de la POP; (c) pacientes que reciben tratamiento no quirúrgico en el ámbito ambulatorio o en el hogar. Se utilizaron los siguientes criterios de exclusión: (a) revisiones, guías, protocolos, resúmenes y ponencias de congresos, editoriales, etc.; (b) literatura no china ni en inglés; (c) publicaciones repetidas, literatura retirada; (d) literatura cuyo texto completo no estaba disponible; (e) mujeres que se sometieron a cirugía o tenían previsto someterse a ella.

### Procedimiento de Búsqueda

Se llevó a cabo una búsqueda sistemática en nueve bases de datos electrónicas, entre ellas PubMed, Web of Science, Embase, CINAHL, Cochrane Library, China National Knowledge Infrastructure (CNKI), Wanfang Database, VIP Database y la Chinese Biomedicine Literature Database (CBM-SinoMed). La búsqueda abarcó el período desde el inicio de las bases de datos hasta el 16 de enero de 2025. Los términos de búsqueda incluyeron Pelvic Organ Prolapse/Urogenital Prolapse/Vaginal Vault Prolapse/Rectal Prolapse/Uterine Prolapse/Cystocele/ Visceral Prolapse” “Patient Reported Outcome Measures/ Patient reported outcome/patient health questionnaire/ selfreport”. (Prolapso de órganos pélvicos/Prolapso urogenital/ Prolapso de la cúpula vaginal/Prolapso rectal/Prolapso uterino/ Cistocele/Prolapso visceral y Medidas de resultados informadas por el paciente/Resultados informados por el paciente/ Cuestionario de salud del paciente/Autoinforme). La estrategia de búsqueda detallada para PubMed se presenta en la Tabla 1.

**Tabla 1:** Estrategias de búsqueda en las bases de datos de PubMed Mianyang, Sichuan, China, 2025.

PubMed	#1:”Pelvic Organ Prolapse”(MeSH Terms) OR “Pelvic Organ Prolapse”(Title/Abstract) OR “urogenital prolapse”(Title/ Abstract) OR “vaginal vault prolapse”(Title/Abstract) OR “rectal prolapse”(Title/Abstract) OR “uterine prolapse”(Title/ Abstract) OR “Cystocele”(Title/Abstract) OR “visceral prolapse”(Title/Abstract) #2:”Patient Reported Outcome Measures”(MeSH Terms) OR “Patient Reported Outcome Measures”(Title/Abstract) OR “patient reported outcome”(Title/Abstract) OR “patient health questionnaire”(Title/Abstract) OR “self-report”(Title/Abstract) #3: #1 ADN #2

### Selección y Extracción de la Bibliografía

Todos los artículos recuperados se gestionaron mediante EndNote 20. Tras eliminar los duplicados, dos investigadores revisaron de forma independiente los títulos y los resúmenes basándose en criterios de elegibilidad predefinidos, a lo que siguió una revisión del texto completo. Cualquier desacuerdo surgido durante el proceso de selección se resolvió mediante el debate con un tercer investigador. Se utilizó un formulario estandarizado de extracción de datos para recolectar información de forma independiente, incluyendo el autor, el año de publicación, el país, el tamaño de la muestra, el diseño del estudio, la población del estudio, el instrumento PRO, el contenido del instrumento, los campos de aplicación, el formulario de evaluación, la frecuencia de evaluación y el entorno de evaluación. A continuación, se resumieron y analizaron los datos extraídos.

## RESULTADOS

### Búsqueda Bibliográfica

La búsqueda inicial en la base de datos arrojó 1548 artículos, todos los cuales se importaron a EndNote 20. Tras eliminar los duplicados, se conservaron 1127 artículos. A continuación, estos artículos se seleccionaron por título y resumen, lo que condujo a la exclusión de 1069 artículos. Los 78 artículos restantes se sometieron a una selección del texto completo, tras lo cual se excluyeron 39. Finalmente, 19 estudios cumplieron los criterios de inclusión y se incluyeron en esta revisión, como se muestra en la Figura 1.

**Figura 1:** PRISMA flowchart of search strategy results – Mianyang, Sichuan, China, 2025.

### Características de la Investigación

Las fechas de publicación de la bibliografía incluida abarcan desde 2014 hasta 2024, y consisten en 18 artículos en inglés y 1 artículo en chino. Actualmente, la investigación en este campo está dispersa geográficamente, centrándose principalmente en los países desarrollados, con un total de 11 artículos publicados. Los países en desarrollo han publicado un total de 8 artículos. Existe variedad en el diseño de la investigación, que incluye principalmente 10 ensayos controlados aleatorios, 6 estudios prospectivos, 2 estudios cuasiexperimentales y 1 estudio retrospectivo de casos y controles. Las características básicas de la bibliografía incluida se muestran en la Tabla 2.

**Tabla 2:** Descripción de los artículos seleccionados, según autor, año, país, diseño del estudio, población del estudio, tamaño de la muestra, contenido de los PRO, instrumentos de PRO, campos de aplicación, formulario de evaluación, frecuencia de evaluación y entorno de evaluación - Mianyang, Sichuan, China, 2025.

Autor	Año	País	Diseño del estudio	Población del estudio	Tamaño de la muestra	Contenido de los PRO	Instrumentos de EPR	Campos de aplicación	Formulario de evaluación	Frecuencia de la evaluación	Entorno de evaluación
Hagen et al.^(16)^	2016	Reino Unido, N ueva Zelanda	Ensayos controlados aleatorizados multicéntricos	Mujeres de cualquier edad con POP en estadio 1-3	412 (grupo de control = 207/grupo de intervención = 207)	Prolapso, función sexual, síntomas vesicales e intestinales y calidad de vida	POP-SS PISQ4R SF-12 ICIQ-UI-SF ICIQ-B	Evaluación de la eficacia clínica	Cuestionarios en papel enviados por correo	Al inicio del estudio, 1 y 2 años después de la aleatorización	Consultas de ginecología en los centros participantes
Hagen et al.^(17)^	2014	Reino Unido, N ueva Zelanda, Australia	Ensayos controlados aleatorizados multicéntricos	Mujeres con prolapso en estadio 1-3	447 (grupo de control = 22 / grupo de intervención = 225)	Prolapso, función sexual, síntomas vesicales e intestinales y calidad de vida	POP-SS PISQ-IR SF-12 ICIQ-UI-SF ICIQ-B	Evaluación de la eficacia clínica	Correo postal o teléfono	Al inicio del estudio, 6 y 12 meses después de la inscripción	Consultas de ginecología en los centros participantes
Strohbehn et al.^(18)^	2024	Estados Unidos	Estudios prospectivos	Pacientes con prolapso en estadio >2 según el POP-Q	78	Prolapso, función sexual, vejiga, síntomas intestinales, dolor, satisfacción	PFDI-20 PFIQ-7 PISQ-IR Satisfacción global EVA	Evaluación de la eficacia clínica	Presencial en papel o por teléfono	1 mes al inicio del estudio y 3 meses durante el periodo de intervención (visitas 0, 1 y 3)	Centro de ensayos clínicos de urogineco logia
Mao et al.^(l9)^	2018	China	Estudios prospectivos	Paciente con una bandeja uterina con anillo de soporte colocada con éxito	142	Prolapso, vejiga, síntomas intestinales, imagen corporal	PFDI-20 PFIQ-7 PGI-C	Evaluación de síntomas/ Calidad de vida	Presencial en papel o por teléfono	Seguimiento medio de 1 7 meses (rango de 13 a 24 meses), momentos de seguimiento (meses 3, 6, 1 2 y cada 6 meses a partir de entonces)	Consultas externas de obstetricia y ginecología
Bugge etal.^(20)^	2024	Gran Bretaña	Ensayo controlado aleatorizado	Pacientes que utilizaron soportes uterinos durante al menos dos semanas	340 (grupo de control 1 69/grupo de intervención 171)	Síntomas del suelo pélvico (prolapso, vejiga, intestino), función sexual, calidad de vida, autoeficacia	PFIQ-7 PFDI-20 EQ-5D-5L GSES PISQ-IR PGI-I	Evaluación de la eficacia clínica/ Calidad de vida	Cuestionario enviado por correo, teléfono, entrevista presencial, enlaces electrónicos	Total: 18 meses; inicio del estudio; seguimiento a los 6, 12 y 1 8 meses	Maternidad hospitalaria o consulta externa
Meriwether et al.^(21)^	2015	Estados Unidos	Estudios prospectivos	Pacientes con soportes uterinos colocados	127	Función sexual, imagen corporal	PISQ-IR mBIS	Calidad de vida	Presencial en papel	Inicio, tres meses	Clínica de tratamiento de la bandeleta uterina
Ai et al.^(22)^	2018	China	Estudios prospectivos	Pacientes que utilizan soportes uterinos	102	Prolapso, Intestino, Vejiga, Calidad de vida, Depresión	PFDI-20 PFIQ-7 PHQ-9	Evaluación de síntomas/ Calidad de vida	Papel presencial	Inicial, 3 meses	Clínica del Centro del Suelo Pélvico
Ouch et al.^(23)^	2018	Japón	Estudios cuasi-experimentales	Pacientes con prolapso en estadio 2 o 3	27	calidad de vida	P-QOL Calidad de vida específica del POP	Calidad de vida/ Evaluación de los síntomas	Presencial en papel o por teléfono	Inicial (antes del PFMT), al final del PFMT de 4 meses, seguimiento a los 2 años	Departamento de Cirugía Urológica o domicilio
Gorji et al.^(24)^	2019	Irán	Ensayo controlado aleatorizado	Mujeres con POP-Q en estadio 2 o 3	40 (20 en el grupo de intervención/20 en el grupo de control)	Síntomas del tracto urinario inferior, calidad de vida	ICIQ-FLUTS P-QOL	Evaluación de la eficacia clínica	Sin informar	Inicio del estudio, 4 semanas después de la intervención	Clínica de Urología
Pizarro-Berdichevsky etal.^(25)^	2016	Chile	Estudios prospectivos	Pacientes en estadio 2 y superior	91	Calidad de vida, depresión	PFDI-20 P-QOL PISQ-12 GHQ-12	Evaluación de síntomas/ Calidad de vida	Sin informar	Inicio del estudio, tras 1 2 meses de seguimiento	Clínica de uroginecología
Limbutara et al.(26)	2023	Tailandia	Ensayo controlado aleatorizado	Mujeres con POP-Q en estadio 2 o 3	40	Calidad de vida, función sexual, objetivos del tratamiento	GAS Escala VAS para la consecución de los objetivos del tratamiento PISQ-IR P-QOL	Evaluación de la eficacia clínica	Papel presencial	Inicio del estudio, 2 semanas y 6 semanas después del tratamiento	Clínica de uroginecología
Crowle and Harley^(27)^	2020	Gran Bretaña	Estudio retrospectivo de casos y controles	Pacientes tratados de forma conservadora	23	Prolapso vesical, síntomas intestinales	PFDI-20	Evaluación de la eficacia clínica	Presencial en papel	Inicial, tras el último tratamiento (duración media del tratamiento: 3 meses)	Hospitales privados o clínicas de fisioterapia
Sweta et al.^(28)^	2018	India	Ensayo controlado aleatorizado	Pacientes con POP leve	50 (grupo de intervención 25/ grupo de control 25)	Prolapso, vejiga, síntomas intestinales	PFDI-20 PFIQ-7	Evaluación de la eficacia clínica	Papel presencial	Inicio del estudio, semanas 4 y 8 tras la intervención, y al final de la semana 12	Sin informar
Shobeiri and Santiagoi^(29)^	2014	Estados Unidos	Ensayo controlado aleatorizado	Pacientes con prolapso en estadios 1 a 3	447 (grupo de intervención 225/ grupo de control 222)	Síntomas del prolapso	POP-SS	Evaluación de la eficacia clínica	Sin informar	Al inicio del estudio, a los 6 y a los 12 meses	Sin informar
Wiegersma etal.^(30)^	2014	Países Bajos	Ensayo controlado aleatorizado	Pacientes de edad avanzada en estadio 1 y 2	287 (grupo de intervención 145/ grupo de control 142)	Percepción del prolapso, vejiga, intestino, función sexual, calidad de vida, cambios sintomáticos	PFDI-20 PFIQ-7 PISQ-12 SF-12 EVA para la evaluación del efecto del tratamiento	Evaluación de los síntomas	Cuestionario presencial en papel/por correo	Inicio del estudio, 3 meses después de la intervención	Sin informar
Wiegersma et al.^(31)^	2014	Países Bajos	Ensayo controlado aleatorizado	Pacientes de edad avanzada en estadio 2 y 3	447 (grupo de intervención 226/ grupo de control 221)	Prolapso, vejiga, intestino, disfunción sexual, calidad de vida, carga sintomática	PFDI-20 PFIQ-7 PISQ-12 SF-12 EQ-5D	Evaluación de la eficacia clínica	Cuestionario presencial en papel/por correo	Al inicio del estudio, 3 meses, 12 meses y 24 meses después del tratamiento	Sin informar
Min et al.^(32)^	2023	China	Ensayo controlado aleatorizado	Pacientes con prolapso en estadios 1-3	11 7 (grupo de control 58/grupo de intervención 59)	Prolapso, vejiga, intestino, función sexual, calidad de vida, ansiedad, depresión, autoeficacia	PFDI-20 PISQ-12 SAS SDS QLQ-52 GSES	Evaluación de la eficacia clínica	Presencial en papel	Inicio del estudio, 3 meses después de la intervención	Consultas externas de hospitales
Ziv and Erlich^(33)^	2022	Israel	Estudio cuasi-experimental	Mujeres con prolapso en estadio 2-4 según la clasificación POP-Q	52	Prolapso, vejiga, intestino, calidad de vida	PFDI-20 PFIQ-7 Puntuación de alivio de los síntomas del POP	Evaluación de la calidad de vida/ eficacia clínica	Presencial en papel o por teléfono	Inicio del estudio, 2 años después del uso	Visitas a la clínica
Coelho etal.^(34)^	2018	Brasil	Estudios prospectivos	Pacientes con prolapso en estadio 3-4	19	Síntomas vaginales, sexualidad, calidad de vida	SF-36 ICIQ-VS	Calidad de vida/ Evaluación de síntomas	Papel, presencial	Inicio del estudio, 6 meses después del uso	Clínica de ginecología

PFDI-20: Inventario de molestias del suelo pélvico-20; PFIQ-7: Cuestionario de impacto del suelo pélvico-7; PISQ-IR Cuestionario sobre incontinencia urinaria: y prolapso de órganos pélvicos Incontinencia Urinaria Cuestionario Sexual; ICIQ-UI-SF: ICIQ-Formulario abreviado de incontinencia urinaria; ICIQ-FLUTS: Cuestionario de la Consulta Internacional sobre Incontinencia: Síntomas del tracto urinario inferior femenino; ICIQ-B: Cuestionario de la Consulta Internacional sobre Incontinencia: Vejiga; PISQ-12: Cuestionario de función sexual y incontinencia-I2; ICIQ-VS: Cuestionario de la Consulta Internacional sobre Incontinencia: Síntomas Vaginales; POP-SS: Puntuación de Síntomas de Prolapso de Órganos Pélvicos; Puntuación de alivio de síntomas de POP: Puntuación de Prolapso de Órganos Pélvicos autoadministrada Informe de Síntomas de Prolapso autoadministrado; POP-QOL: Calidad de vida específica para el prolapso-QOL: Cuestionario de calidad de vida en el prolapso; SF-12: Cuestionario abreviado de 12 ítems; QLQ-52: Calidad de vida-52; SF-36: MOS Cuestionario abreviado de salud de 36 ítems; EQ-5D-5L: EuroQol de 5 dimensiones, cinco niveles; EQ-5D: EuroQol-5D Cuestionario; SAS: Escala de autoevaluación de la ansiedad; SDS: Escala de autoe valuación de la depresión; PHQ-9: Cuestionario de salud del paciente-9; GHQ-12: Cuestionario de salud de Goldberg de 12 ítems; VAS: Escala analógica visual; mBIS: Escala modificada de imagen corporal femenina; CAS: Escala de consecución de objetivos; PGI-I: Impresión global del paciente sobre la mejoría; PGI-C: Impresión

### Instrumentos PRO y Contenido

En los 19 estudios incluidos se identificaron un total de 29 instrumentos PRO aplicados en el tratamiento no quirúrgico del prolapso de órganos pélvicos (POP). La población objetivo de estas herramientas estaba compuesta principalmente por pacientes con prolapso en estadios I-III según la clasificación POP-Q, y los dominios evaluados en todos los instrumentos incluían la valoración de los síntomas, el estado psicológico, la calidad de vida y el logro de objetivos. Para la evaluación de los síntomas, los instrumentos utilizados incluyeron el Inventario de Malestar del Piso Pélvico-20 (PFDI-20), el Cuestionario de Impacto del Piso Pélvico-7 (PFIQ-7), el Cuestionario Sexual sobre Prolapso de Órganos Pélvicos/Incontinencia Urinaria (PISQ-IR), el Formulario Corto de Incontinencia Urinaria del ICIQ (ICIQ-UI-SF), el Cuestionario de la Consulta Internacional sobre Incontinencia: Síntomas del Tracto Urinario Inferior en Mujeres (ICIQ-FLUTS), el Cuestionario de la Consulta Internacional sobre Incontinencia: Vejiga (ICIQ-B), el Cuestionario de Función Sexual y Incontinencia-12 (PISQ-12), Puntuación de Síntomas de Prolapso de Órganos Pélvicos (POP-SS), una Puntuación de Prolapso de Órganos Pélvicos autoadministrada (para el alivio de los síntomas), un Informe de Síntomas de Prolapso autoadministrado y el Cuestionario de la Consulta Internacional sobre Incontinencia: Síntomas Vaginales (ICIQ-VS). Para la evaluación de la calidad de vida, los instrumentos identificados fueron el Cuestionario de Calidad de Vida en el Prolapso (P-QOL), la Encuesta de Forma Corta de 12 ítems (SF-12), el Cuestionario de Calidad de Vida-52 (QLQ-52), Cuestionario de Salud de 36 Items (SF-36), EuroQol-5 Dimensiones-5 Niveles (EQ-5D-5L) y el Cuestionario EuroQol-5D (EQ-5D). En cuanto al estado psicológico, las escalas utilizadas incluyeron la Escala de Autoevaluación de la Ansiedad (SAS), la Escala de Autoevaluación de la Depresión (SDS), el Cuestionario de Salud del Paciente-9 (PHQ-9), el Cuestionario de Salud de Goldberg de 12 ítems (GHQ-12) y la Escala de Imagen Corporal Femenina modificada (mBIS). Por último, las herramientas relacionadas con la consecución de objetivos y la satisfacción con el tratamiento comprendieron la Escala de Consecución de Objetivos (GAS), una Escala Analógica Visual para la Consecución de Objetivos Terapéuticos, la Impresión Global del Paciente sobre la Mejora (PGI-I), la Impresión Global del Paciente sobre el Cambio (PGI-C), la Escala General de Autoeficacia (GSES) y una Escala Analógica Visual para la Satisfacción Global.

### Características de los Instrumentos PRO

Entre los 29 instrumentos PRO incluidos en este estudio, a excepción de 2 que no presentan una distinción dimensional clara, el resto de los instrumentos PRO se componen de entre 1 y 9 dimensiones. El número de ítems por herramienta varió ampliamente, oscilando entre 1 y 52, y el método de puntuación principal fue la escala de Likert. La mayoría de los estudios incluidos eran investigaciones de seguimiento que requerían que los pacientes se adhirieran a un tratamiento a largo plazo. Solo un pequeño subgrupo de pacientes que recibían autocuidado no necesitaba acudir al hospital para evaluaciones presenciales por parte del personal médico durante el seguimiento. En consecuencia, la recolección de datos se llevó a cabo principalmente en los servicios de atención ambulatoria de los hospitales, en centros de fisioterapia y en entornos comunitarios. En cuanto a la forma de recolección de los cuestionarios, la recolección de cuestionarios en papel sigue siendo la forma principal.

De los 19 estudios, 3^(24,25,29)^ no informaron en detalle sobre la forma de recolección de los cuestionarios de resultados reportados por el paciente (PRO), y 8^(16,22,26,27,28,31,32,34)^ utilizaron un único método de recolección mediante cuestionarios en papel. Los 8 estudios restantes emplearon una combinación de métodos, incluyendo cuestionarios telefónicos y por correo. Cabe destacar que solo un estudio^(20)^ utilizó cuestionarios electrónicos. Este estudio también mencionó el suministro de información de contacto y formatos de respuesta múltiple para adaptarse a las preferencias de los pacientes, ofreciendo así un enfoque de evaluación más flexible. Un total de 17 estudios^(16,17,18,18,19,20,21,22,23,24,25,26,27,28,30,31,32,33,34)^ util izaron múltiples instrumentos de PRO simultáneamente para medir los síntomas, la calidad de vida, la satisfacción y el estado psicológico de los pacientes. Los otros 2 estudios^(27,29)^ con el tema de la mejora de los síntomas del prolapso utilizaron un único PRO para la medición de un solo atributo de la gravedad de los síntomas de las pacientes, pero uno de estos estudios contaba con la herramienta en sí misma como una herramienta de medición de múltiples atributos, capaz de reflejar la gravedad de los síntomas de las pacientes, la calidad de vida, etc.

## CAMPOS DE APLICACIÓN DE LOS INSTRUMENTOS PRO

### Evaluación de la Eficacia Clínica

El Comité Asesor Internacional de Uroginecología recomienda^(35)^ que los PRO validados se utilicen como estándar de referencia en la evaluación de los síntomas del POP, junto con el examen clínico cuantitativo del prolapso de órganos pélvicos (POP-Q). Por lo tanto, el uso de los PRO como indicador de evaluación para el tratamiento clínico de los pacientes es una de las direcciones de aplicación más importantes en el campo del tratamiento no quirúrgico del POP. De los 19 estudios incluidos, 12 implicaron el uso de los PRO como medida de resultado para la evaluación de los resultados del tratamiento. Estos estudios se centraron principalmente en pacientes con prolapso en los estadios I-III del POP-Q y evaluaron el manejo de la salud de las pacientes en el hogar o en entornos ambulatorios, así como los efectos de diversas intervenciones no quirúrgicas. Las medidas evaluadas incluyeron ejercicios personalizados del suelo pélvico, el uso de pesarios novedosos, la implementación de modelos de enfermería basados en teorías de autoeficacia y actividades de ejercicio físico como el yoga y las posturas. Los indicadores evaluados se centraron principalmente en los síntomas relacionados con el POP, incluyendo el prolapso, la micción anormal, las deposiciones anormales y las alteraciones sexuales. Además, también se incluyeron en el estudio otros indicadores, como la satisfacción de las pacientes con el efecto del tratamiento, la confianza en el tratamiento, el cumplimiento de los objetivos del tratamiento y la adherencia.

### Calidad de Vida

Aunque el POP es una afección no mortal, tiene una alta tasa de incidencia, implica costos de tratamiento sustanciales y afecta significativamente la salud física y mental y la calidad de vida de las mujeres afectadas^(36)^. La calidad de vida relacionada con la salud, que se refiere al estado de salud general de una persona y al bienestar subjetivo en los ámbitos físico, mental y social, es un constructo clave evaluado por instrumentos PRO en diversos contextos relacionados con la atención médica^(37)^. Por lo tanto, el uso de herramientas PRO para evaluar la calidad de vida en pacientes sometidas a tratamiento no quirúrgico del POP representa otro importante enfoque de investigación en este campo. Ocho de los 19 estudios^(19,20,21,22,23,25,33,34)^ evaluaron la calidad de vida de las pacientes. La mayoría de los estudios utilizaron instrumentos genéricos de calidad de vida para los trastornos del suelo pélvico, mientras que solo cinco^(23,24,25,26,34)^ emplearon la escala P-QOL, un cuestionario específico para el POP. En los estudios restantes, aunque la calidad de vida no era el enfoque principal, los instrumentos PRO utilizados incluían dominios relacionados con este constructo. Esto refleja un esfuerzo más amplio por comprender de manera integral cómo los tratamientos no quirúrgicos mejoran el funcionamiento diario y el bienestar general en pacientes con POP.

### Evaluación de los Síntomas

El objetivo principal del tratamiento de la POP es aliviar los síntomas. Por lo tanto, el uso de instrumentos PRO para evaluar y manejar los síntomas físicos y psicológicos o el funcionamiento durante el tratamiento no quirúrgico es un área clave de investigación.

De los 19 estudios incluidos, 6^(14,22,23,25,30,34)^ evaluaron el funcionamiento mental y físico en pacientes sometidos a tratamiento no quirúrgico.

Estos estudios evaluaron una amplia gama de síntomas en diversos ámbitos. Tres estudios^(19,23,34)^ se centraron exclusivamente en los síntomas físicos, siendo el abultamiento y la presión pélvica los más comúnmente reportados. Además, tres estudios^(22,25,30)^ evaluaron los síntomas físicos junto con otros resultados, en lugar de centrarse en ellos como criterios de valoración principales. El uso de instrumentos de resultados informados por el paciente (PRO) para evaluar los síntomas psicológicos en pacientes con POP también fue un tema clave de investigación. Tres estudios^(18,22,25)^ evaluaron los síntomas psicológicos, un número relativamente pequeño. Sin embargo, todos fueron estudios prospectivos de gran tamaño, y dos^(22,25)^ examinaron específicamente la relación causal entre los síntomas depresivos y el POP. Otros síntomas psicológicos evaluados incluyeron la ansiedad, la angustia y las deficiencias funcionales relacionadas.

### Momentos de Evaluación de los Instrumentos PRO

Cada uno de los 19 artículos incluidos en este estudio informó los momentos de evaluación de los instrumentos PRO en pacientes con POP que recibían tratamiento no quirúrgico. Aunque los momentos para la recolección de datos de los PRO variaron entre los estudios, todos los estudios incluidos realizaron una evaluación inicial antes de la intervención o del inicio del tratamiento. El número de evaluaciones mediante cuestionario por paciente osciló principalmente entre 2 y 5 veces. La mayoría de los estudios^(21,22,24,25,27,30,32,34)^ realizaron dos evaluaciones, consistentes en la cumplimentación de cuestionarios de PRO antes y después de la intervención no quirúrgica. Además, 5 estudios^(19,20,23,28,31)^ realizaron evaluaciones de PROs más de 3 veces. Todos ellos fueron estudios de seguimiento con un período de seguimiento relativamente largo, que osciló entre 2 semanas y 2 años, y el número de evaluaciones aumentó a medida que se prolongaba la duración del seguimiento. Entre ellos, los momentos de recolección de datos de los PRO se concentraron principalmente a los 3, 6 y 12 meses, que se utilizan ampliamente para evaluar los efectos a medio y largo plazo de las intervenciones. Algunos estudios se diseñaron para monitorear los cambios en los resultados del tratamiento durante el seguimiento domiciliario entre pacientes que recibían tratamiento no quirúrgico. Estos estudios tenían diseños flexibles y duraciones de seguimiento variables, que podían incluir evaluaciones previas y posteriores a la intervención, así como evaluaciones de seguimiento periódicas.

## DISCUSIÓN

### Gran Heterogeneidad de los Instrumentos de PRO en el Campo del Tratamiento no Quirúrgico de la POP

Actualmente, existen amplias variaciones en los tipos de PRO, el contenido, el número de ítems y los momentos de evaluación utilizados en el campo del tratamiento no quirúrgico de la POP. Existe una amplia variedad de PRO disponibles para evaluar los síntomas en pacientes sometidos a tratamiento no quirúrgico de la POP. Entre los incluidos en esta revisión, los más utilizados fueron el PFDI-20, el PFIQ-7 y el PISQ-IR. Sin embargo, debido a las limitaciones en la adaptación lingüística e intercultural, las tasas de validación y aplicación de algunas escalas siguen siendo bajas en diferentes países y regiones^(11)^. Esto dificulta en cierta medida la promoción de herramientas de EPR en el ámbito internacional del tratamiento no quirúrgico del POP y la comparación de estudios transnacionales de alta calidad. Además, dos estudios^(23,33)^ utilizaron cuestionarios de evaluación de síntomas de diseño propio para el tratamiento no quirúrgico de pacientes con POP, pero los procedimientos y pasos de la preparación de los cuestionarios no se describieron en los estudios, y su fiabilidad, validez y representatividad deben verificarse más a fondo. Por lo tanto, los esfuerzos futuros deben hacer hincapié en la traducción rigurosa, la adaptación transcultural y la validación de las herramientas existentes. En segundo lugar, los 29 PRO incluidos en esta revisión variaron considerablemente en su contenido dimensional, abarcando múltiples dominios. En cuanto a las prioridades de evaluación para las pacientes con POP, Sung et al.^(38)^ describieron constructos clave, entre ellos: (1) alivio de los síntomas de protuberancia vaginal, (2) mejora del funcionamiento físico, (3) mejora del funcionamiento sexual, (4) mejora de la percepción de la imagen corporal y (5) mejora del funcionamiento social.

Sin embargo, ninguno de los PRO incluidos en esta revisión abarcó todos estos constructos, lo que limitó la exhaustividad de las evaluaciones en pacientes sometidas a tratamiento no quirúrgico del POP. Además, la elección de los momentos de evaluación de los PRO varió entre los estudios. Once estudios evaluaron a las pacientes al inicio, después de la intervención y en el seguimiento, con intervalos que oscilaron entre semanas y años. Cuatro estudios^(18,19,23,31)^ emplearon un enfoque estratificado por etapas para la recolección de datos. La heterogeneidad en los momentos de evaluación puede haber contribuido a la variabilidad en los hallazgos de los estudios. Investigaciones previas^(35)^ han señalado una falta de datos longitudinales sobre la progresión, la resolución y el tratamiento de la POP. En consecuencia, la importancia clínica de la intervención temprana y el momento óptimo para el tratamiento siguen sin estar claros. La variabilidad en el momento de la evaluación de los PRO puede ser un factor que dificulte la comprensión integral de las trayectorias de los síntomas y los resultados del tratamiento en pacientes con POP no quirúrgicos. Además, algunos estudios administraron múltiples PRO simultáneamente, lo que condujo a un mayor número de ítems o a una superposición conceptual, aumentando así la carga para los pacientes^(39)^. Por lo tanto, los esfuerzos futuros deben centrarse en el desarrollo de herramientas de PRO completas y concisas para pacientes con POP no quirúrgicos, a fin de captar con precisión las características y necesidades de los pacientes. Al mismo tiempo, existe una necesidad urgente de establecer un calendario de evaluación estandarizado basado en las características de las diferentes herramientas de PRO, mejorando así el sistema de evaluación de PRO para pacientes con POP no quirúrgicos.

### Es Necesario Mejorar el Modelo de Monitoreo de Salud digital de los PRO en el Campo Del Tratamiento no Quirúrgico de la POP

Los instrumentos de PRO suelen ser completados por los pacientes y pueden administrarse a través de diversos formatos, incluyendo cuestionarios en papel, entrevistas telefónicas, cuestionarios enviados por correo y encuestas electrónicas. Los diferentes formatos de PRO requieren una validación por separado^(40)^. Los resultados indican que la recolección actual de datos de PRO en el tratamiento no quirúrgico del POP se basa principalmente en cuestionarios en papel solos o en una combinación de cuestionarios telefónicos y por correo. Estudios previos^(41)^ han demostrado que los métodos tradicionales de recolección de datos en papel no solo son menos eficientes, sino que también son propensos a problemas como datos incompletos y pérdida de información. En esta revisión, solamente Bugge et al.^(20)^ utilizaron cuestionarios electrónicos para recolectar datos de PRO. Sin embargo, el estudio no realizó una validación psicométrica adicional de la versión electrónica, lo que puede afectar la confiabilidad y validez de sus hallazgos. Gray et al.^(42)^ encontraron que las medidas electrónicas de PRO (ePRO) pueden reducir la carga de los investigadores en la recolección y gestión de datos, mejorar la eficiencia y precisión del procesamiento de datos, y permitir a los pacientes completar las evaluaciones en casa antes de su visita inicial. Esto permite a los médicos acceder a información clave del paciente con anticipación, optimizando así el proceso de diagnóstico y tratamiento. Además, el ePAQ es rentable, ya que permite una recolección de datos más rápida y reduce significativamente tanto el tiempo de gestión como los costos de implementación^(43)^. Actualmente, el ePAQ-Pelvic Floor (ePAQ-PF) es el único cuestionario electrónico específico para la POP que cuenta con una validación sólida^(44)^. Sin embargo, no se ha utilizado en pacientes que reciben tratamiento exclusivamente no quirúrgico para el POP y, por lo tanto, no se incluyó en esta revisión. Por lo tanto, las investigaciones futuras deberían centrarse en el desarrollo de herramientas electrónicas de PRO específicas para pacientes con POP no quirúrgicos, integrando diversas tecnologías de salud digital para mejorar la experiencia del paciente y abordar diversas necesidades de investigación. Además, al convertir los cuestionarios en papel existentes a formatos electrónicos, es esencial verificar sus propiedades psicométricas para garantizar la validez y fiabilidad de las nuevas versiones.

### Aún es Necesario Reforzar la Aplicación en Tiempo de los PRO Para Evaluar los Cambios DinÁmicos en los Síntomas de los Pacientes con POP no Quirúrgicos

A medida que la atención médica se orienta hacia un modelo holístico y centrado en el paciente, el uso de los PRO en pacientes sometidos a tratamiento no quirúrgico para el POP cobra cada vez más importancia^(45)^. Los estudios han demostrado que los PRO deben incorporarse a la práctica clínica diaria y a la investigación, ya que facilitan la comunicación entre pacientes y médicos y respaldan el desarrollo de estrategias de tratamiento óptimas^(46)^. Esta revisión encontró que las herramientas de EPR se utilizan principalmente en pacientes con POP no quirúrgico para evaluar los síntomas, la calidad de vida y los efectos clínicos de las intervenciones no quirúrgicas. La investigación sobre la evaluación de los síntomas sigue siendo limitada, centrándose principalmente en la mejoría de los síntomas después de la intervención o en las correlaciones entre el POP y otros síntomas, prestando poca atención al seguimiento a largo plazo de los cambios dinámicos de los síntomas. Sin embargo, los estudios han demostrado^(47)^ que los síntomas en pacientes con POP no son estáticos, sino que fluctúan con el tiempo.

Algunos de los estudios incluidos en esta revisión^(22,25,30,34)^ se centraron únicamente en los cambios de los síntomas en el momento inicial y en los puntos finales de seguimiento, pasando por alto las fluctuaciones dinámicas de los síntomas. Esto puede haber llevado a los investigadores a sobreestimar o subestimar la eficacia de la intervención y haber obstaculizado el análisis en profundidad de los mecanismos que vinculan los síntomas del POP y las afecciones relacionadas. Además, la mayoría de las pacientes prefieren un calendario de evaluación flexible cuando utilizan herramientas de EPR, lo que les permite proporcionar retroalimentación oportuna a los proveedores de atención médica y recibir intervenciones específicas cuando no se sienten bien^(48)^. Los estudios existentes se han centrado principalmente en la presentación de informes estructurados de EPR a intervalos fijos durante el tratamiento y el seguimiento del POP^(49)^. Sin embargo, este enfoque puede dar lugar a una subnotificación o a un retraso en la notificación de los síntomas, lo que disminuye el valor de los EPR para el monitoreo dinámico. Por lo tanto, los estudios futuros deberían prestar mayor atención a los cambios dinámicos de los síntomas en pacientes sometidos a tratamiento no quirúrgico del POP, con el fin de captar más plenamente las experiencias en tiempo real de los pacientes y proporcionar una base más precisa para la toma de decisiones clínicas. Al mismo tiempo, la optimización del proceso de evaluación y el monitoreo en tiempo real de los pacientes representan direcciones importantes para la investigación futura en este campo.

### Los Resultados de los Informes de PRO se han Convertido Gradualmente en un Indicador de Resultados Importante

El Instituto Nacional para la Salud y la Excelencia Clínica (NICE)^(50,51)^ del Reino Unido recomienda el tratamiento no quirúrgico como opción de primera línea para todas las pacientes con POP. Por lo tanto, medir los resultados del tratamiento no quirúrgico es esencial para el manejo posterior de las pacientes. Estudios previos han evaluado principalmente los resultados del tratamiento en pacientes con POP utilizando indicadores clínicos objetivos. Sin embargo, estos indicadores tienen limitaciones a la hora de evaluar la eficacia de los resultados del tratamiento. Por un lado, los estudios^(52,53)^ han demostrado que los cambios anatómicos en el POP no siempre se correlacionan con la gravedad de los síntomas. En consecuencia, aunque los indicadores objetivos, como la posición de los órganos y la estadificación sintomática, puedan mejorar tras el tratamiento, es posible que la percepción de los síntomas por parte de las pacientes, su calidad de vida o su satisfacción con el tratamiento no mejoren al mismo tiempo. Por otro lado, factores como las experiencias cotidianas de las pacientes, su estado psicológico y su funcionamiento social pueden influir significativamente en su experiencia de la enfermedad y en su capacidad para afrontarla. Estas percepciones subjetivas, dinámicas y multidimensionales no pueden captarse plenamente mediante métodos objetivos como el examen pélvico o las técnicas de imagen^(38)^. Por lo tanto, los PRO son cruciales para evaluar el tratamiento no quirúrgico del POP y reflejan un enfoque de la atención centrado en la paciente. Los estudios incluidos en esta revisión utilizaron herramientas cuantitativas de PRO para informar sobre los resultados en pacientes sometidas a tratamiento no quirúrgico del POP. De los 12 estudios que evaluaron los resultados clínicos, ocho utilizaron los resultados de los PRO como su medida de resultado principal^(16,17,18,20,24,26,29,31)^. Los expertos en el campo del suelo pélvico y los comités pertinentes^(54)^ abogan por incluir los resultados de los PRO en los resultados del tratamiento del POP, una recomendación coherente con los hallazgos de esta revisión. Por último, cabe señalar que los datos de resultados de salud reportados por los pacientes a partir de los PRO son un componente importante de los resultados clínicos. Sin embargo, los resultados de los PRO deben integrarse con indicadores de laboratorio clínico, medidas de resultados reportadas por el médico (ClinROM) y otros datos de resultados de múltiples fuentes para reflejar de manera integral la salud del paciente y evaluar los efectos de las intervenciones clínicas^(55)^.

## CONCLUSIÓN

Los PRO se han utilizado en el tratamiento no quirúrgico de la POP para evaluar los resultados clínicos, la calidad de vida y la valoración de los síntomas, principalmente para medir el grado de mejoría en los síntomas de los pacientes, incluida la evaluación de su calidad de vida y los síntomas psicológicos. Sin embargo, algunos estudios no han realizado un seguimiento de los cambios dinámicos en los síntomas de los pacientes, lo que ha dado lugar a una falta de supervisión y manejo individualizados de los pacientes. En el futuro, se deben establecer estándares uniformes para la frecuencia y el intervalo de las evaluaciones, y se debe crear un mecanismo de seguimiento dinámico para monitorear mejor los cambios sintomáticos de los pacientes, al tiempo que se debe utilizar la tecnología digital para mejorar la conveniencia y la eficiencia de la evaluación. Además, se deben ampliar los campos de aplicación de los PRO y se debe fortalecer la evaluación integral del estado psicológico de los pacientes, y se debe potenciar enérgicamente la cooperación multidisciplinaria y la colaboración internacional para promover el desarrollo de un modo de manejo individualizado basado en los PRO.

Sin embargo, una limitación clave de esta revisión es la exclusión de los estudios que no están en inglés ni en chino. Esta decisión puede haber limitado la generalización de nuestros hallazgos e introducido un sesgo lingüístico. Por lo tanto, las revisiones futuras deberían considerar la inclusión de una gama más amplia de idiomas para garantizar una comprensión más integral y culturalmente diversa de las aplicaciones de los PRO en el tratamiento de la POP.

## Data Availability

Todo el conjunto de datos que apoya los resultados de este estudio fue publicado en el propio artículo.
